# A preliminary study of mirror-induced self-directed behaviour on wildlife at the Royal Belum Rainforest Malaysia

**DOI:** 10.1038/s41598-020-71047-1

**Published:** 2020-08-24

**Authors:** Azwan Hamdan, Mohd Qayyum Ab Latip, Hasliza Abu Hassim, Mohd Hezmee Mohd Noor, Tengku Rinalfi Putra Tengku Azizan, Noordin Mohamed Mustapha, Hafandi Ahmad

**Affiliations:** 1 Department of Veterinary Preclinical Sciences, Faculty of Veterinary Medicine, University Putra Malaysia, 43400 UPM Serdang, Selangor Darul Ehsan Malaysia; 2 Department of Veterinary Pathology and Microbiology, Faculty of Veterinary Medicine, University Putra Malaysia, 43400 UPM Serdang, Selangor Darul Ehsan Malaysia; 3 Laboratory of Sustainable Animal Production and Biodiversity, Institute of Tropical Agriculture and Food Security, University Putra Malaysia, 43400 UPM Serdang, Selangor Darul Ehsan Malaysia; 4 University Agriculture Park, Universiti Putra Malaysia, 43400 UPM Serdang, Selangor Darul Ehsan Malaysia

**Keywords:** Physiology, Ecology

## Abstract

Mirror-induced behaviour has been described as a cognitive ability of an animal to self-direct their image in front of the mirror. Most animals when exposed to a mirror responded with a social interactive behaviour such as aggressiveness, exploratory and repetitive behaviour. The objective of this study is to determine the mirror-induced self-directed behaviour on wildlife at the Royal Belum Rainforest, Malaysia. Wildlife species at the Royal Belum Rainforest were identified using a camera traps from pre-determined natural saltlick locations. Acrylic mirrors with steel frame were placed facing the two saltlicks (*Sira Batu* and *Sira Tanah*) and the camera traps with motion-detecting infrared sensor were placed at strategically hidden spot. The behavioural data of the animal response to the mirror were analysed using an ethogram procedure. Results showed that barking deer was the species showing the highest interaction in front of the mirror. Elephants displayed self-directed response through inspecting behaviour via usage of their trunk and legs while interacting to the mirror. Interestingly, the Malayan tapir showed startled behaviour during their interaction with the mirror. However, the absence of interactive behaviour of the Malayan tiger signalled a likelihood of a decreased social response behaviour. These results suggested that the ability to self-directed in front of the mirror is most likely related to the new approach to study the neural mechanism and its level of stimulus response in wildlife. In conclusion, research on mirror-induced self-directed behaviour in wildlife will have profound implications in understanding the cognitive ability of wildlife as an effort to enhance the management strategies and conservation.

## Introduction

Mirror-induced behaviour in animals provides some evidence for self-directed behaviour, which is described as the ability of animals to interact or respond their image in front of the mirror. Study on the mirror-induced self-directed behaviour may increase on cognitive challenge and stimulus response behaviour^1–4^. In addition, animals produced self-directed behaviour by the used of mirror reflection may enable an enhanced individual ability to be aware of its new environment^5^. The used of mirror to determine the animal’s interaction has been discovered by Gallup who pioneered in the mirror self-recognition (MSR). Ideally, the study suggested that MSR goes through four stages of behaviour when animals react to the image from the mirror viz; social response, physical mirror inspection, repetitive mirror-testing behaviour and realization of seeing themselves or self-recognition^1,6^. It has been suggested that the final stage of MSR is verified when an animal has successfully recognized the mark on their head ^1,6,7^. However, the application of the mark test depends on the several factors such as brain size^6^, location of the mark^8^ and whether the animals are in captivity or in the wild^6^. Hence, there exist difficulties in putting an identifiable mark on free roaming wildlife species.

The development of mirror-induce self-directed behaviour in animals has been linked with physiological, social contexts and response outcome during learning tasks^9,10^. However, most of animals exposed to a mirror responded with social interactive behaviour such as aggression, investigation and repetition against the reflection^1–4,11^. The step by step behavioural response as reported in previous studies showed that most animals with high cognition such as primates will perceive mirror image as a living being by displaying social interaction^11,12^. The animal will then progress to self-body inspection after abandoning any form of social behaviour towards the mirror image. These are deemed as “contingency” reaction whereby the animal will elicit repetitive physical movement such as head swaying, hand waves or even whole body movements^13^. Theoretically, animals responded to their reflection in one of three ways: (i) animals behave in front of the mirror due to the conspecific or another animal, and show aggressive behaviour towards it^1–3^; (ii) animals recognize the image as an illusory and eventually ignore it^6,14^; and (iii) animals recognize themselves in front of the mirror and starts to self-directed interaction^4,7^.

Abundant mirror-induced self-directed behaviour studies have been conducted in captive and solitary animals such as chimpanzees^1,2,11,12^, dolphins^8^, elephants^7,15^, ants^16^, magpies^4^, fish^17^ and crows^18^. However, there is a limited scientific data on mirror-induced behaviour studies in the wild due to the difficulties to identify the individual animal, hence difficulties to score different behavioural observations for the same or different individual. Therefore, we hypothesized that some of the wildlife; (i) are interested in the mirror reflection especially at the start of the experiment, (ii) are not interested with the image, (iii) would show social behaviour towards the mirror image, and (iv) would not show self-directed behaviours. Thus, this study will be exploring the mirror-induced self-directed behaviour of different wildlife species in the Royal Belum Rainforest, Malaysia. This reserved and protected forest located in Gerik Perak, Malaysia houses majority of the fauna species including the barking deer or muntjac (*Muntiacus muntjak*), sambar deer (*Rusa unicolor*), Malayan Sun bear (*Helarctos malayanus*), seladang (*Bos gaurus*), Asian elephant (*Elephas maximus*), and the Malayan tiger (*Panthera tigris jacksoni*)^19^. The diversities and species richness especially at the natural saltlick of the rainforest makes it a suitable environment to discover mirror-induced self-directed behaviour especially in wildlife. Furthermore, the study of mirror-induced self-directed behaviour in wildlife is based on aspect of physiological and behavioural functions of animals, and how this mechanism correlated with social and ecological interactions towards enhancing the management strategies for the endangered species in the rainforest.

## Materials and methods

### Materials

The equipment used in this study was a one-way acrylic mirror (150 × 120 cm) made from a highly durable and anti-shatter material and was supported by a 45 cm pole above the ground by a wooden stand (Fig. 2). The size of the mirror in this preliminary study is chosen as an indicator to determine the species of wildlife presence and interact in front of the mirror. Three camera traps (Bushnell, USA) with red light motion detector were used to record any movement or interaction of animals in front of the mirror. The camera recorder was set up to take a short 20 s video at 5 min intervals. Alkaline batteries and 24 megabyte of memory space were used for the camera trap, which were estimated to last for up to 2 months. The permit of wildlife research and Royal Belum Rainforest entry permits were approved by the Department of Wildlife Malaysia and Perak State Park, Malaysia.

### Sampling location

**Figure 1:**
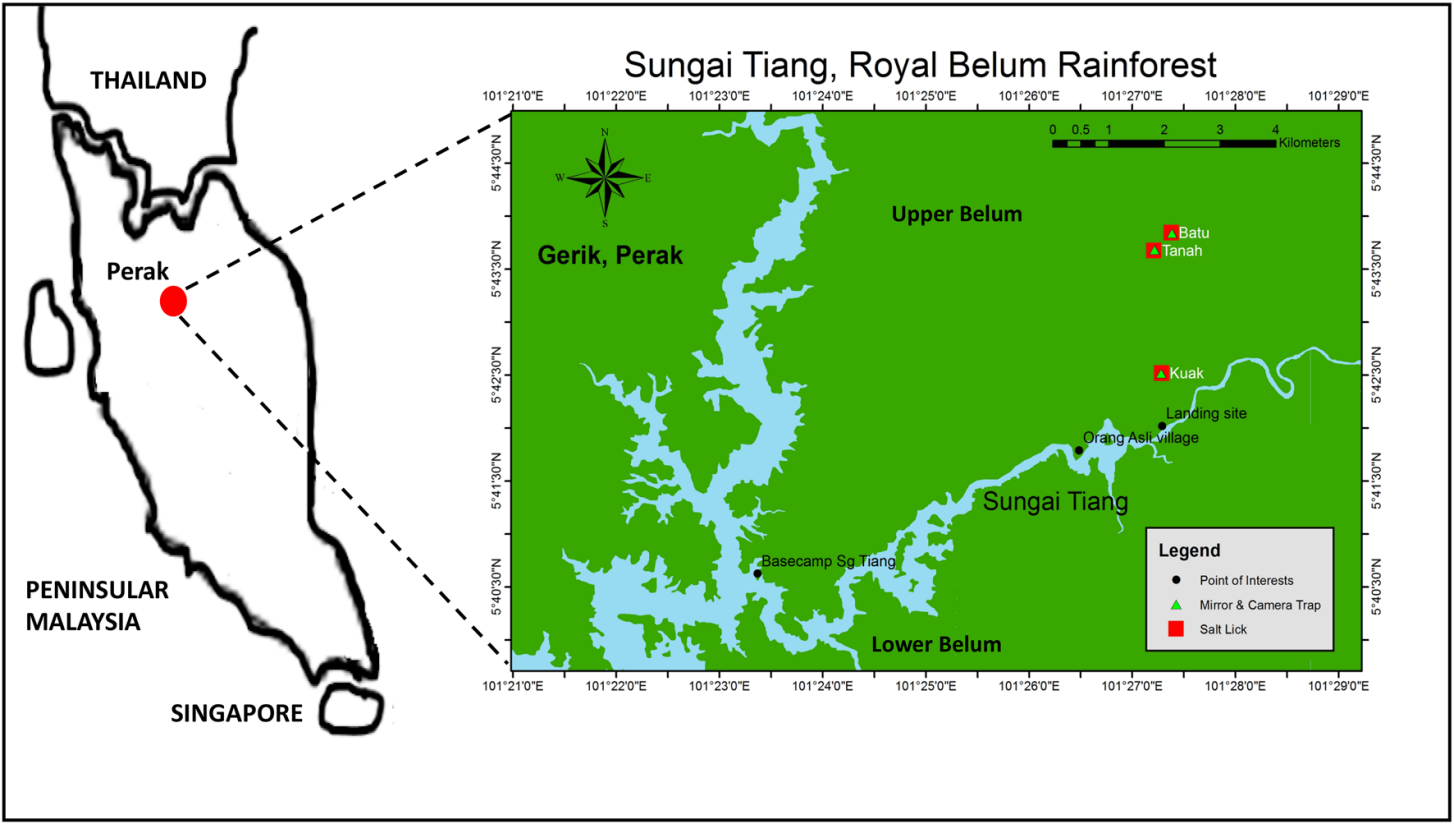
The location of Sungai Tiang and Royal Belum Rainforest, Gerik, Perak Malaysia. Map details: (1) Peninsular Malaysia: The map was illustrated by the author of this manuscript. (2) Sungai Tiang, Royal Belum Rainforest: Generated from Google Earth (https://www.google.com/earth/) and ArcGIS (https://www.esri.com/en-us/arcgis/products/arcgis-desktop/overview) software application with GPS reference coordinate; 5° N latitude and 101° E longitude.

The study was conducted from January 2017 to January 2019 at the Royal Belum Rainforest, Gerik Perak Malaysia. Figure 1 shows the location of the study area geographically situated at 5° N latitude and 101° E longitude. This protected area was gazetted under the Park State Corporation Enactment 2001 by the Perak State Government on 17 April 2007. This gazetted rainforest is divided into two sections; the upper Belum area which stretches to the Malaysia-Thailand border covering an area of 117,500 hectares, and the lower Belum area which is about 300,000 hectares.

There are diversities in the study area with lowland and hill dipterocarp tropical forest types such as impenetrable jungles and natural saltlick. The natural saltlick has been identified through animal trail and animal foot print near the saltlick. The natural saltlick are chosen as study site due to high chances of wildlife presence for mineral requirement for the animals^20,21^. Two natural saltlicks have been identified such as *Sira Batu* and *Sira Tanah*. *Sira Batu* spanned over about 52 m^2^ and located about 2 h hike away from the *Sungai Tiang* base camp (Fig. 1). *Sira Batu* is unique in a sense that its front portion is facing a small river with a stony structure. The other three sides were enclosed by a slight embankment with a minimal incline. The area had large trees with some shrubbery within the vicinity of a bamboo forest where the saltlick is easily accessible to all species.

*Sira Tanah* only spans an area of 20 m^2^ and located about 2 h hike from the landing point and closed to *Sira Batu* which is about 15 min hike away. There is a steep embankment on one side of the saltlick and a vast flatland with minimal plants extending about 30 m around the saltlick. There is a small stream located in front of the saltlick that drains from one end into the stream. This saltlick is also easily accessible for all species with ample space for herding wildlife species.

### Experimental procedures

**Figure 2:**
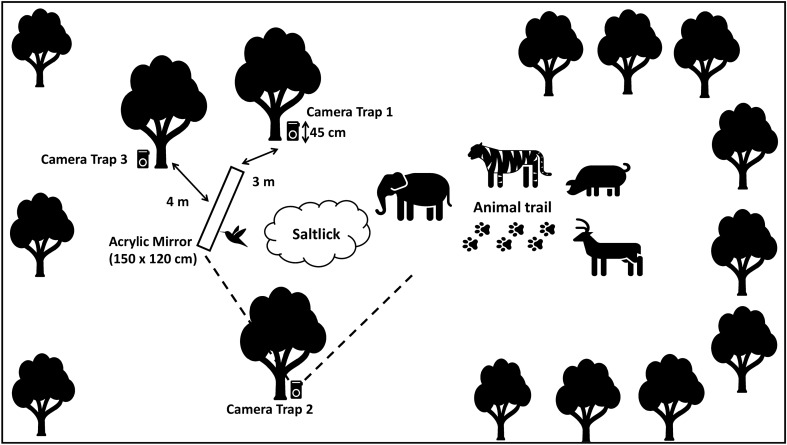
The location of the mirror and the camera trap set-up at the saltlick area. The illustrations was designed by the author of this manuscript.

Figure 2 shows the location of the mirror and the camera traps at the saltlick areas. Three camera traps were placed near the saltlick area and animal trail. The camera trap Number 1 was placed within 3 m from the mirror and at a height of 45 cm from the ground, roughly followed the average height of most wildlife species. The camera trap Number 2 was placed to capture the whole area of the saltlick and the mirror. The camera trap Number 3 was placed within 4 m from the back of the mirror. The camera was placed at a sufficient distance in order to capture the whole image and footage. In addition, the camera has the capability to capture wide angle image so that the animals can be documented as a whole.

The camera traps were placed at the saltlick area for approximately 2 months depending on the frequencies of the video captured or based on movement image triggers. The batteries and memory card were replaced to a new set of batteries and memory card intermittently within a 2 year duration of study. All data from the memory card were played back with Microsoft Windows media player and the behaviours of the wildlife such as barking deer, Malayan tapir, birds species, Malayan tiger and elephants were tabulated on ethogram table.

### Data analysis

Data obtained from the study were analyzed with SPSS 17.0 for Windows (SPSS Inc., Chicago, USA) using one-way analysis of variance (ANOVA). The data was tabulated as mean ± SEM and only *P* less than 0.05 was considered as significant.

## Results and discussion

**Figure 3:**
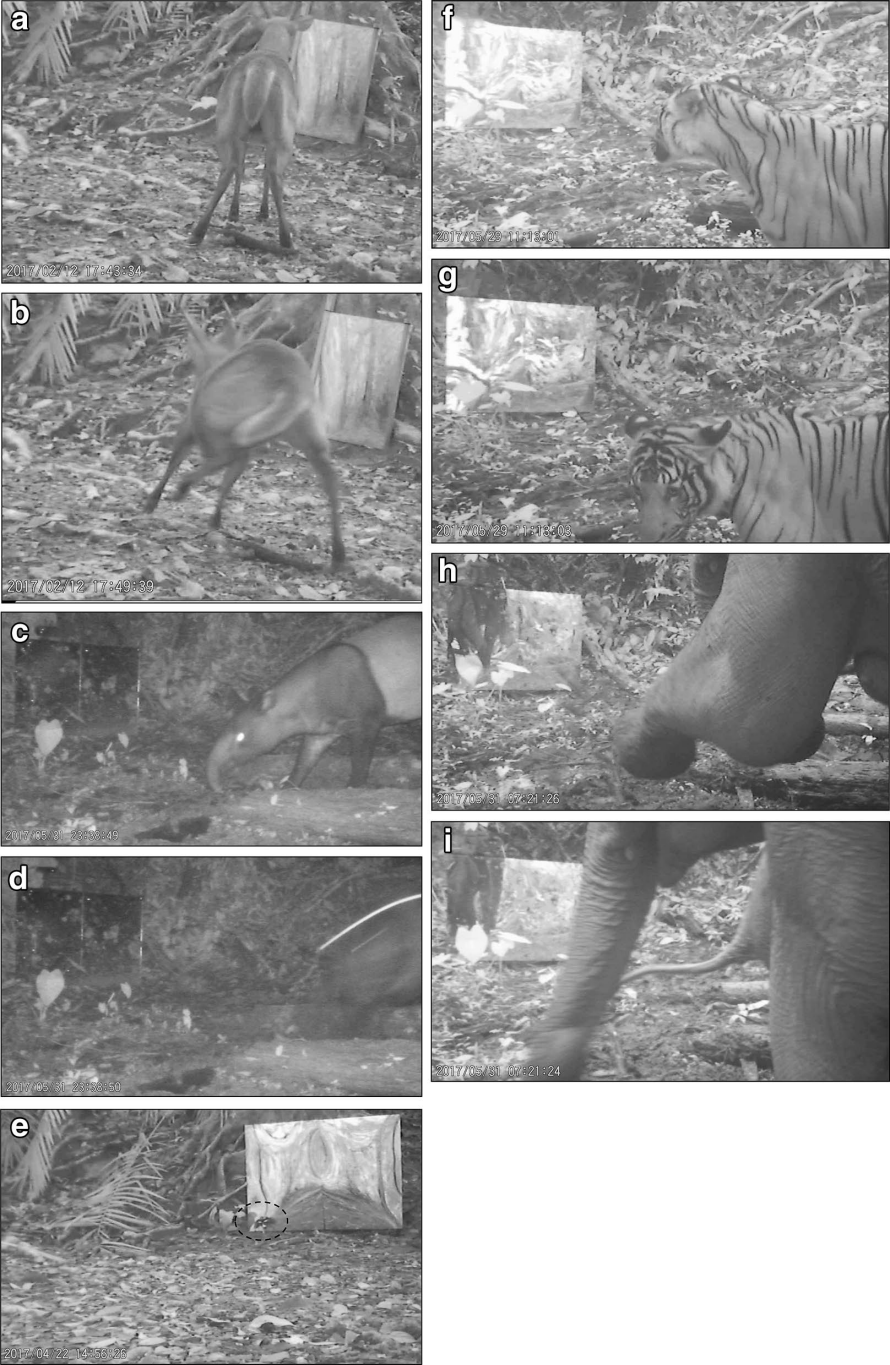
(**a**) Barking deer spent time at looking at the mirror with (**b**) movement or stumping of their legs. (**c**) Behavior of Malayan tapir before recognized the reflection from the mirror. (**d)** Malayan tapir displayed startled behaviour. (**e**) The bird showed wing-flashing and jumping towards the mirror, most likely as fighting or attacking to the image. (**f**) Malayan tigers showed spent approximately 3 s with the eyes target to the mirror. (**g**) Eventually, the tiger disregard the reflection or image. (**h**) A group of elephants used their leg, and (**i**) trunk to interact with their self-image in front of the mirror.

The frequencies and the image of mirror-induced self-directed behaviours according to species have been described in the Table 1 and Fig. 3, respectively. The barking deer is the species that exhibited the highest (*P* < 0.05) interaction in front of the mirror (463.0 ± 129.70 frequencies), followed by the birds (296.0 ± 54.70 frequencies) and tapir (105.0 ± 16.4 frequencies).

**Table 1:** The frequencies and types of mirror-induced self-directed behaviour of wildlife at the Royal Belum Rainforest, Malaysia.

Species	Types of behaviour in front of the mirror	Frequencies (mean ± SE)
(1) Barking deer	Staring for two or more seconds	17.0 ± 10.3
	Shocking (startled) and running away	4.0 ± 0.8
	Looking at the mirror	178.0 ± 41.9*
	Moving its head	64.0 ± 18.3
	Walking towards the mirror	10.0 ± 4.1
	Walking and startled by its reflection and running away	7.0 ± 0.4
	Stumping both front legs	47.0 ± 13.2*
	Stumping leg	85.0 ± 10.6*
	Change its body towards the mirror	7.0 ± 2.9
	Walking away and ignoring the mirror	7.0 ± 2.7
	Investigating the mirror structure	1.0 ± 0.6
	Stand still in front of the mirror	2.0 ± 0.9
	Investigating the reflection from the sunlight	2.0 ± 0.6
	Looking at the mirror and walking away	3.0 ± 0.6
	Searching food near the mirror	1.0 ± 0.7
	Sniffing the area of saltlick near the mirror	3.0 ± 0.7
	Staring/looking in front of the mirror and chewing food	2.0 ± 0.5
	Fighting with other animal (same species)	1.0 ± 0.2
	Put its head at lower position	5.0 ± 1.1
	Looking behind the mirror	7.0 ± 3.2
	Bending its body	1.0 ± 0.6
	Turning its body towards the mirror	4.0 ± 0.8
	Lifting up its body	1.0 ± 0.1
	Distancing while looking at the mirror	1.0 ± 0.2
	Turn backward	1.0 ± 0.2
	Jumping	1.0 ± 0.1
	Foot scraping	1.0 ± 0.3
Total behaviour of barking deer		463.0 ± 129.7*
(2) Bird species	Looking at the mirror	134.0 ± 22.4*
	Attacking the mirror	107.0 ± 17.9*
	Walking at the side of the mirror	7.0 ± 3.5
	Pecking the mirror	46.0 ± 10.4*
	Flying towards the mirror	1.0 ± 0.3
	Walking in front of the mirror	1.0 ± 0.2
Total behaviour of bird species		296.0 ± 54.7*
(3) Malayan tapir	Looking at the mirror and running (startled)	18.0 ± 7.4
	Sniffing and touching the mirror by using its snout	13.0 ± 5.1
	Staring at the mirror	3.0 ± 0.8
	Walking towards the mirror	18.0 ± 5.3
	Looking at the mirror	9.0 ± 7.2
	Moving backward	15.0 ± 7.5
	Running away	24.0 ± 11.2
	Sniffing the area near the mirror	5.0 ± 6.4
Total behaviour of Malayan tapir		105.0 ± 16.4*
(4) Asian elephant	Looking at the mirror	43.0 ± 12.3
	Moving its trunk and leg in front of the mirror	16.0 ± 8.4
	Pull back its front right leg	5.0 ± 0.5
	Moving its front right leg	9.0 ± 1.0
	Turn it's body to the left	5.0 ± 0.4
	Moving backward	6.0 ± 0.5
	Scrape the ground in front of the mirror	3.0 ± 0.1
	Walking away from the mirror	2.0 ± 0.3
Total behaviour of Asian elephant		89.0 ± 23.5
(5) Sambar deer	Staring at the mirror	4.0 ± 0.7
	Walking towards the mirror	5.0 ± 0.6
	Investigating the reflection	1.0 ± 0.3
	Moving its head towards the mirror	24.0 ± 9.4
	Sniffing the mirror	1.0 ± 0.2
	Moving backward	2.0 ± 0.9
	Looking at the mirror	38.0 ± 12.3
Total behaviour of sambar deer		75.0 ± 24.4
(6) Malayan tiger	Looking at the mirror	25.0 ± 11.8
	Walking in front of the mirror	3.0 ± 0.2
Total behaviour of Malayan tiger		28.0 ± 12.0
(7) Mousedeer	Staring/looking in front of the mirror	3.0 ± 0.2
	Running away from the mirror	1.0 ± 0.2
Total behaviour of mousedeer		4.0 ± 0.4
(8) Eagle	Looking at the mirror	2.0 ± 1.0
(9) Rat	Sniffing behind the mirror	4.0 ± 2.0
(10) Porcupine	Sniffing behind the mirror	2 ± 0.4
(11) Common wild pig	Looking at the mirror	1.0 ± 0.2
(12) Monkey	Investigating the mirror	1.0 ± 0.3

*Significantly different at *P* < 0.05.

The barking deer spent most of the time at looking at the mirror (178.0 ± 41.9; Table 1) followed by movement or stumping of their legs in front of the mirror (85.0 ± 10.6; Fig. 3a,b; Video 1). This possibly indicated that the barking deer could not recognize itself in front of the mirror via investigating its reflection. This higher social interaction of barking deer found in the study presented here suggested that an understanding the evolution of its behaviour is more advanced and highly socialise in ungulates^22^. The used of mirror as an enrichment would connectively stimulate the self-directed behaviours of barking deer. It has been reported that ungulates shows a great diversity in spacing patterns of their home range, social structure and mating systems^23^. In fact, the barking deer is a solitary forest dwelling species inhabiting dense tropical and sub-tropical forest of Asia, which are considered by some as primitive in the deer family^24^.

Interestingly, Malayan tapir displayed startled behaviour upon looking or staring at the mirror and eventually ran away from the mirror (18.0 ± 7.4, Table 1; Fig. 3c,d; Video 2). The startled behaviour could be due to the animal response towards the reflection that being other or similar species present during the saltlick visit. This behaviour has been associated with motor functions that normally occurs in wildlife animals; for instance when they interacted with the reflection in water^25^. Physiologically, these rapid response to the reflected image is due to the behavioural response to perceived threats or aversi stimuli^26^. In this study, the video output showed that the tapirs tend to be solitary when exploring the environment and seems to be active mostly at night as previously suggested^27,28^. This is indeed a well-known fact that wildlife is often easily distracted and displayed coping behaviour even in the situation of minor irregularities in the environment such as sudden changes in temperature, physical restraint and threats from conspecifics or the approach of a human^29,30^.

In birds, the highest frequencies was recorded in looking in the mirror (134.0 ± 22.4; Table 1) followed by the attacking the mirror (107.0 ± 17.9; Fig. 3e; Video 3). Most of the time, exploration of the bird was characterized by approaches towards the front and back of the mirror. The birds also moved their head and the whole body back and forth the mirror in a systematic way. It has been reported that birds such as magpie showed a short period of intense activity in front of the mirror, indicating contingency testing^4^. Under social interaction behaviour, birds showed wing-flashing and jumping towards the mirror, most likely as an offensive move towards the image (Fig. 3e). This possibly indicated that they examine the extend of the mirror image being coupled to their own movement. However, as seen in mammals, the birds will disregard the image or reflection following familiarization with the mirror. This study also indicated that the higher frequencies of attempting themselves with the mirror is linked with the self-directed behaviour, which could enhance the evolution of social and cognitive intelligence in bird species^18^.

In our study, Malayan tigers interacted in front of the mirror by a looking behaviour (25.0 ± 11.8; Table 1) with eyes targeting the mirror for approximately 3 s and eventually disregarded their own reflection (Fig. 3f,g; Video 4). Based on the results, there is a low physical interaction of the Malayan tiger in front of the mirror. This could either be that the tiger recognizes mirror reflection image or thought that the reflection was that of another animal. Our data also suggested that tigers recognized their mirror image as conspecific, rather than as illusion of themselves. However, previous study have been reported that some feline species may treat their image with playing, fighting or confronting with the mirror and ending up completely confused^31^. There are several factors that influences the discrepancy of mirror-induced self-directed behaviour findings on the Malayan tiger especially in Royal Belum Rainforest. This may include extinction of the population primarily due to habitat loss or poaching leading the tigers to being ignorant of others^32^ and their natural solitary behaviour in a wide range of forest^33,34^.

A group of elephants in Royal Belum Rainforest was able to interact with their self-image in front of the mirror by using their trunk and leg (Fig. 3h,i; Video 5). This finding is similar to that described by Plotnik et al*.* where captive elephants touch most part of their own body with their trunk in front of the mirror. In this study, the elephant showed higher frequencies on the self-directed behaviour by looking (43.0 ± 12.3; Table 1) and interacting at the mirror using trunk and leg (16.0 ± 8.4; Table 1). This could indicate that the wild elephants showed self-behavioural flexibility and adaptability in social groups towards the reflection. Although wild elephants live in large home range, the social system remains to be complex with effects from external factors such as food availability, competition for space and human presence. These factors allow for adaptability between the groups of elephants that would influence the behavioural response to the mirror image in close proximity. In addition, the elephants in the wild are quick to respond with the enrichments or objects such as mirror and camera trap that would contribute a strangest or unfamiliar to them^7,15,35^. In fact, numerous images captured in this study showed that elephants are furiously inspecting the camera traps.

There are several factors that influenced the variation of the mirror-induced self-directed behaviours in the wildlife species. Our findings suggested that the mirror-induced self-directed behaviours in wildlife are correlated with the brain developmental and maturity of the species, which is quite challenging when it comes to identifying the age of animal in the wild. Moreover, the study of mirror-induced self-directed behaviour is based on different brain neurological system such as the level of encephalization (EQ) in animals^8,36,37^. Previous studies reported that the level of EQ is associated with the size of the brain of animals^7,38,39^. Indeed, the size of the brain is an informative index of evolved brain and self-recognition^38^. Physiologically, the self-directed behaviour of animals are linked to the brain superior longitudinal fasciculus, which a brain system might mediate the mirror-induced behaviours^12^. Thus, this could indicate that the behaviour responses to a mirror provides compelling evidence for convergent cognitive evolution and mental state attribution in wildlife species.

The capacity of mirror-induced self-directed behaviours in wildlife are the beginning of a developmental process of self-recognition towards its new environment or enrichment. An investigation of mirror-induced self-directed behaviour in wildlife is not only of interest regarding the behaviour reaction of animals towards the mirror but it is also providing a valuable understanding of the general ecosystem in the forest that influenced the cognitive, social skills and intelligence of animals. To what extent the self-directed behaviour are developed with increasing exposure to a mirror from wildlife species is still unknown, however some wildlife developed the self-directed behaviour through the ponds or water ditches^25^. Therefore, further studies need to be carried out to explore the capacity of mirror-induce behaviour in the wildlife. In fact, the animals only developed the self-directed behaviour in front of the mirror during the early sampling of study and nevertheless animals start to treat the mirror as a conspecific or just disregard it when the mirror is placed at long periods at the saltlick area. Furthermore, there are challenges in performing the mirror-induced self-directed behaviour in wild animals due to several factors such as the size and the strength of the acrylic mirror if attacked by large animals. We will continue using the data from this study for specific wildlife behaviour in future studies using a larger sized mirror. Other factors to be included and monitored are humidity of the rainforest environment and the durability of the camera trap batteries. Sometimes, the behaviour of wildlife is affected by the infra-red illumination from the camera, even though the camera were placed at a hidden spot^40^.

## Supplementary information

Supplementary Information.
