# Supplementary material for: A preliminary study of mirror-induced self-directed behaviour on wildlife at the Royal Belum Rainforest Malaysia
Source: Sci Rep. 2020 Aug 24;10:14105. doi: 10.1038/s41598-020-71047-1 (PMC7445253; doi:10.1038/s41598-020-71047-1)
Supplement: Supplementary file 1 — Supplementary Information. [file 41598_2020_71047_MOESM1_ESM.docx]

**A preliminary study of mirror-induced self-directed behaviour on wildlife at the Royal Belum Rainforest Malaysia**

**Azwan Hamdan^1^, Mohd Qayyum Ab Latip^1^, Hasliza Abu Hassim^1,3^, Mohd Hezmee Mohd Noor^1, 4^, Tengku Rinalfi Putra Tengku Azizan^1^, Noordin Mohamed Mustapha^2^ and Hafandi Ahmad^1*^**

^1^Department of Veterinary Preclinical Sciences, Faculty of Veterinary Medicine, University Putra Malaysia, 43400 UPM Serdang Selangor Darul Ehsan MALAYSIA

^2^Department of Veterinary Pathology and Microbiology, Faculty of Veterinary Medicine, University Putra Malaysia, 43400 UPM Serdang Selangor Darul Ehsan MALAYSIA

^3^Laboratory of Sustainable Animal Production and Biodiversity, Institute of Tropical Agriculture and Food Security, University Putra Malaysia, 43400 UPM Serdang Selangor Darul Ehsan MALAYSIA

^4^University Agriculture Park, Universiti Putra Malaysia, 43400 UPM Serdang Selangor Darul Ehsan MALAYSIA

*** Corresponding Author:**

Assoc Prof Dr Hafandi Ahmad

Department of Veterinary Preclinical Science

Faculty of Veterinary Medicine

University Putra Malaysia, MALAYSIA

Phone: +603 8609 3416

Email: [hafandi@upm.edu.my](mailto:hafandi@upm.edu.my)

**Supplementary information**

1) Video 1: Barking deer spent time at looking at the mirror with movement or stumping of their legs.

<https://www.youtube.com/watch?v=olSFsV704ok&feature=youtu.be>

2) Video 2: Behaviour of Malayan tapir before recognized the reflection from the mirror and eventually displayed startled behaviour.

<https://www.youtube.com/watch?v=iErvuB7A2QE&feature=youtu.be>

3) Video 3: The bird showed wing-flashing and jumping towards the mirror, most likely as fighting or attacking to the reflection.

<https://www.youtube.com/watch?v=k8Ycw_G3zjc&feature=youtu.be>

4) Video 4: Malayan tigers showed spent approximately 3 seconds with the eyes target to the mirror, and eventually disregarded the reflection.

<https://www.youtube.com/watch?v=jfgCrODnVlk>

5) Video 5: A group of elephants used their leg and trunk to interact with their self-image in front of the mirror.

<https://www.youtube.com/watch?v=9FnYztuu42s&feature=youtu.be>
